# Clavulanic Acid Improves Memory Dysfunction and Anxiety Behaviors through Upregulating Glutamatergic Transporters in the Nucleus Accumbens of Mice Repeatedly Exposed to Khat Extract

**DOI:** 10.3390/ijms242115657

**Published:** 2023-10-27

**Authors:** Amal O. Arab, Fawaz Alasmari, Awatif B. Albaker, Hassan A. Alhazmi, Alaa Alnoor Alameen, Naser M. Alagail, Saleh A. Alwaeli, Syed Rizwan Ahamad, Abdullah F. AlAsmari, Shakir D. AlSharari

**Affiliations:** 1 Department of Pharmacology and Toxicology, College of Pharmacy, King Saud University, Riyadh 11451, Saudi Arabia; 2 Department of Pharmaceutical Chemistry and Pharmacognosy, College of Pharmacy, Jazan University, Jazan 45142, Saudi Arabia; 3 Department of Pharmaceutical Chemistry, College of Pharmacy, King Saud University, Riyadh 11451, Saudi Arabia

**Keywords:** khat, glutamate transporters, clavulanic acid, cathine, cathinone, neurobehaviors

## Abstract

Khat (*Catha edulis*) is an evergreen shrub whose buds and leaves give a state of delight and euphoria when chewed. Cathinone, an amphetamine-like stimulant that is among the active ingredients in khat, is able to downregulate glutamate transporter subtype I (GLT-1). Neurobehavioral dysfunctions such as altered locomotor activity, anorexia, and nociception have been observed in animals exposed to cathinone. Interestingly, treatment with a β-lactam antibiotic such as ceftriaxone, which upregulates GLT-1, normalizes cathinone-induced conditioned place preference, and alters repetitive movements in rats. However, little is known about the role of the glutamatergic system in memory dysfunction and anxiety-like behaviors in mice exposed to khat. We found here that clavulanic acid, a β-lactam-containing compound and GLT-1 upregulator, would modulate the neurobehavioral changes, including memory impairment and anxiety-like behaviors, associated with repeated exposure of mice to khat. Our data supported that clavulanic acid could improve memory impairment and anxiety-like behaviors through upregulating GLT-1 in the nucleus accumbens (NAc), an effect abolished with a selective GLT-1 blocker. This upregulation was associated with restored glutamate/cystine antiporter expression in the NAc using a Western blotting assay. Cathine and cathinone were identified in khat extract using the gas chromatography technique. Our work provides preclinical insight into the efficacy of β-lactam-containing compounds for the attenuation of neurobehavioral changes induced by khat exposure.

## 1. Introduction

Khat (*Catha edulis*) is a seedless plant of the family Celastraceae whose leaves have an aromatic odor and astringent and relatively sweet taste; it can grow in various climates and soils and is cultivated as a bush or a small tree [1]. It is also known as qat, chat, miraa, and Arabian or Abyssinian tea and is cultivated mainly in regions of East Africa, such as Ethiopia and Kenya [2]. The buds and leaves of khat are traditionally chewed, and dried leaves are sporadically infused as tea, but it is only rarely smoked [3]. The major active substances of khat include several alkaloids, tannins, and flavonoids, of which the phenylpropylamine derivative cathinone (S-(−)-α-aminopropiophenone) is the main psychoactive alkaloid, and the phenylpropanolamine diastereomer cathine (S,S-(+)-nor-pseudoephedrine) is a less-psychoactive component. Both affect the central and peripheral nervous systems [4].

The amount of cathinone and cathine in khat leaves is variable. Cathinone is present in higher concentration than cathine in fresh, tender leaves and, over time, is converted to cathine and subsequently norephedrine [5,6]. Many behavioral experiments have been conducted to pharmacologically explain the herbal psychostimulant activity of khat extract or cathinone. Observed behavioral modulations include increased locomotor activity (psychostimulation), anorexia, nociception, behavioral sensitization (psychosis), and aggressive behavior [7,8]. In addition, multiple studies have shown khat to contain a neurotoxic addictive substance with effects on neurobehavioral function [9,10,11,12], with prolonged exposure being linked to psychological dependence and withdrawal symptoms [13].

Glutamate is a primary excitatory neurotransmitter of the central nervous system (CNS) [14,15]. Under physiological conditions, glutamate neurotransmission is achieved via two classes of receptors: ligand-gated ion channels (ionotropic receptors) and metabotropic glutamate receptors (G-protein coupled receptors) [16,17,18]. Emerging evidence supports that astrocytes regulate glutamate uptake via excitatory amino acid transporters (EAATs) [19]. Under normal conditions, glutamate is transported by EAATs into cells, moving against its concentration gradient on account of the co-transport of sodium. Hydrogen ions are also brought into the cell, causing potassium efflux [20]. In this manner, EAATs guarantee rapid and effective removal of glutamate from the synaptic cleft. Impairment of EAAT-mediated glutamate uptake leads to high concentrations of extracellular glutamate, with subsequent cellular injury and glutamate excitotoxicity (described below) [19]. Given their central role in neural glutamate homeostasis, EAAT dysfunction has unsurprisingly been implicated in numerous acute or chronic diseases of the CNS, including ischemic stroke and epilepsy, cerebellar ataxias, amyotrophic lateral sclerosis (ALS), Alzheimer’s disease, and Huntington’s disease [21].

EAAT1 (and its rodent homolog glutamate-aspartate transporter, GLAST) is highly expressed in the cerebellum. EAAT2 (rodent glutamate transporter 1, GLT-1) is the primary glutamate transporter in the forebrain; it is abundant in astrocytes but expressed only to a limited extent in presynaptic nerve terminals [19]. Another astroglial glutamate transporter, cystine/glutamate antiporter (xCT), exchanges glutamate for cystine, thereby transporting it from astrocytes into the presynaptic neurons; communication of xCT with metabotropic glutamate receptors (mGluR2/3) thus plays a critical role in restoring glutamate release capability [19,22]. A previous study showed that astrocytic knockout of GLT-1 was associated with a reduction of a total of 80% of GLT-1 protein expression and glutamate uptake activity in mice, an effect associated with increased mortality and seizures and reduced body weight [23]. No significant changes in mortality rate, seizure occurrence or body weight changes following deletion of GLT-1 in neurons suggest the important role of astrocytic GLT-1 in glutamate uptake and neuroprotection [23].

As mentioned previously, glutamate is rapidly eliminated from the extracellular space under normal conditions through binding and uptake by cell-surface transporters. This rapid removal is essential to ensure controlled and efficient cell-to-cell communication during homeostasis. Astrocytes are primarily responsible for glutamate uptake and catabolize and convert it further into glutamine, then deliver the glutamine back to presynaptic neurons [24]. Given the importance of glutamate uptake to normal function, deficiencies in glutamate uptake are frequently blamed for the initiation or progression of neurological disorders, such as stroke [25]. Additionally, glutamate excitotoxicity is associated with a number of neurological diseases, including the neurodegenerative conditions Parkinson’s disease (PD), Alzheimer’s disease, Huntington’s disease, and ALS [26]. Therefore, glutamate pathways might offer therapeutic targets for these conditions.

Importantly, glutamate pathways are impacted in substance use disorders. Five weeks of ethanol drinking reduces the protein expression of GLT-1 and xCT in central reward brain regions, including the nucleus accumbens (NAc), amygdala, and hippocampus [27,28], and a significant reduction in glutamate uptake has been observed in the NAc of rats exposed to ethanol (1 g/kg i.p.) for seven days [29]. Exposure to cocaine, amphetamine, nicotine, and hydrocodone is associated with decreased GLT-1 and xCT protein expression in the NAc of preclinical models [30,31,32,33]. Likewise, exposure to drugs of abuse is reported to increase extracellular glutamate concentrations [34,35,36]. Notably, the NAc can receive glutamatergic inputs from multiple brain areas [37]. Deficit in glutamate clearance was observed in the NAc, a region attributed to the motivational effects of drugs of abuse [38].

Synthetic cathinone compounds, commonly referred to as “bath salts”, are a popular class of new psychoactive substances. Repeated exposure to synthetic cathinone, 3,4-methylenedioxypyrovalerone (MDPV), followed by withdrawal, has been observed to reduce GLT-1 protein expression in the NAc of rats on day 5 and day 10 later [39]. Accordingly, repeated exposure to MDPV is suggested to modulate GLT-1, which leads to behavioral alterations such as sensitization and locomotor stimulation, effects that can be attenuated with ceftriaxone [39]. However, little is known about the effects of khat extract, more broadly, on memory recognition and anxiety behaviors.

β-lactam antibiotics have demonstrated the ability to attenuate ethanol-, cocaine-, nicotine-, and hydrocodone-seeking behaviors, at least in part by increasing the expression of GLT-1 protein expression in mesocorticolimbic brain regions [30,31,34,40]. Clavulanic acid is a β-lactam drug that functions as a β-lactamase inhibitor but does not possess antibacterial activity and, hence, does not incur antibiotic resistance and other antibiotic-associated side effects. This compound has been shown to attenuate alcohol- and amphetamine-seeking behaviors in part through upregulating GLT-1 and xCT expression in the NAc [33,41]. In the current work, we hypothesized that clavulanic acid could be used to modulate the neurotoxic and neurobehavioral changes associated with repeated exposure to khat extract in mice.

## 2. Results

### 2.1. Average Body Weight

**Figure 1:**
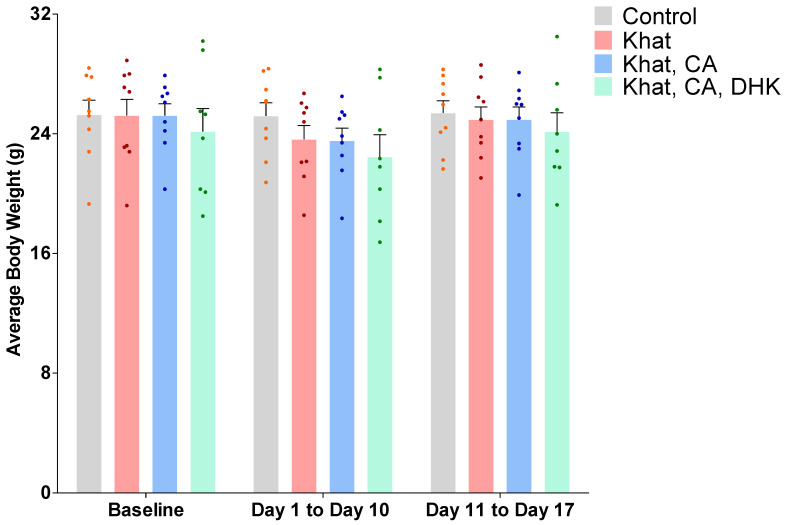
Average body weight at baseline and post-treatment by group. Using two-way ANOVA, there was no significant difference between the groups. *n* = 8–9/group. Color dots represent individual data. CA, clavulanic acid; DHK, dihydrokainic acid; g, gram.

The average body weight of the mice in each group was measured throughout the study period. Using statistical analysis by a two-way ANOVA, we did not reveal any significant differences between the groups, either at baseline or post-treatment for time F (2, 93) = 1.867, for treatment F (3, 93) = 1.353, and for interaction F (6, 93) = 0.1730) (Figure 1).

### 2.2. Behavioral Studies

Locomotor activity was monitored for all groups during the EPM and NORT experiments.

#### 2.2.1. Locomotor Activity Test

**Figure 2:**
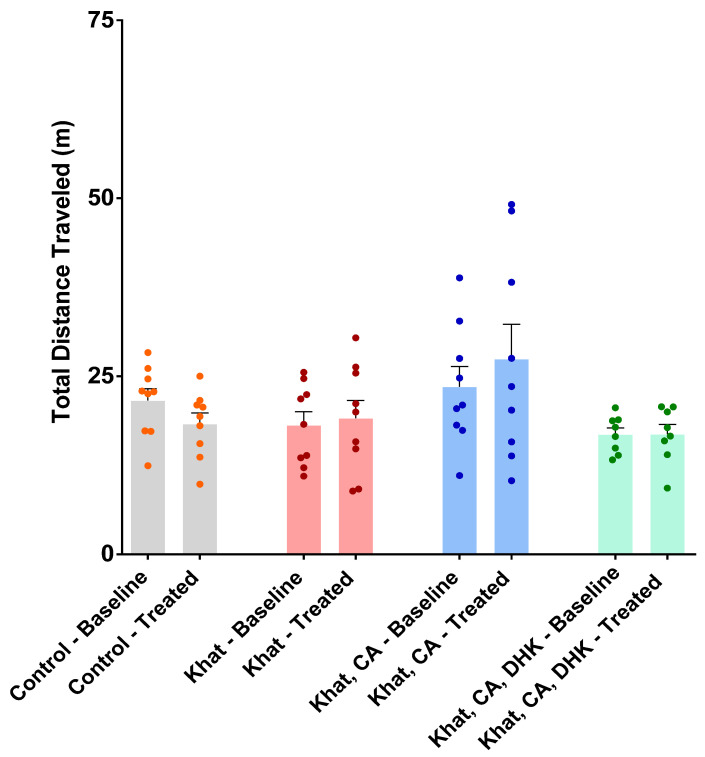
Total distance traveled during the baseline and treatment periods by group. There was no significant within-group difference in locomotor activity using paired *t*-tests. There was no significant between-group difference in locomotor activity using one-way ANOVA. *n* = 8–9/group. Color dots represent individual data. CA, clavulanic acid; DHK, dihydrokainic acid; m, meter.

Locomotor tests were performed to evaluate the function of the musculoskeletal system and the distances mice were inclined to travel. Values from before and after intervention were compared using paired-sample *t*-tests. No statistically significant difference was observed for any of the groups (Figure 2) (Control, t = 1.539 and df = 8; Khat, t = 0.4029 and df = 8; Khat-CA, t = 1.215 and df = 8; Khat-CA-DHK, t = 0.04323 and df = 7). A one-way ANOVA was used to perform a between-group comparison of the mean locomotor activity at baseline and after treatments. Again, no statistically significant difference in locomotor activity in baseline (F (3, 31) = 2.361) and after treatments (F (3, 31) = 2.536) was observed (Figure 2).

#### 2.2.2. Elevated Plus Maze (EPM)

The EPM test was used to evaluate the effects of khat extract and treatments on anxiety-like behaviors. In this test, more time spent in closed arms versus open arms is considered to indicate anxiety.

##### Ratio of Time in Closed Arms

**Figure 3:**
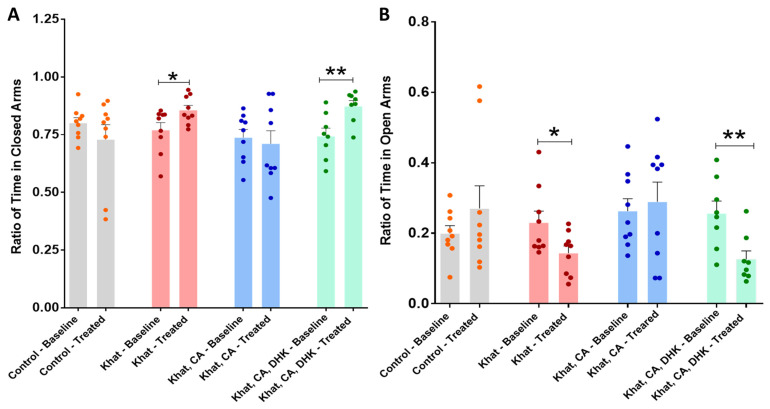
Ratio of time spent in closed and open arms. (**A**) There were significant differences between the pre- and post-intervention ratios of time spent in closed arms for the Khat (* *p* = 0.0354) and Khat-CA-DHK (** *p* = 0.0033) groups using paired *t*-tests. In the same panel, there were statistically significant differences in the post-intervention closed-arm time ratio among the groups using one-way ANOVA, but the differences did not remain significant after applying Tukey’s post hoc test. (**B**) There were significant differences in the pre- and post-intervention ratios of time spent in open arms for the Khat (* *p* = 0.0354) and Khat-CA-DHK (** *p* = 0.0033) groups using paired *t*-tests. In the same panel, there were statistically significant between-group differences in the post-intervention open-arm time ratio using one-way ANOVA, but the differences did not remain significant after applying Tukey’s post hoc test. *n* = 8–9/group. Color dots represent individual data. CA, clavulanic acid; DHK, dihydrokainic acid.

We found statistically significant differences in the pre- and post-intervention closed-arm time ratios within the Khat group (*p* < 0.05) and the DHK-CA-Khat group (*p* < 0.01) using paired *t*-tests (Khat, t = 2.528 and df = 8; Khat-CA-DHK, t = 4.362 and df = 7). For the Control and CA-Khat groups, no significant difference was observed (Figure 3A) (Control, t = 1.193 and df = 8; Khat-CA, t = 0.4397 and df = 8). Although we did not find significant changes in the closed-arm time ratio between groups in the baseline phase using a one-way ANOVA (F (3, 31) = 0.8569), the analysis identified statistically significant between-group differences in closed-arm time ratio only after treatments (F (3, 31) = 3.323, *p* < 0.05). However, these differences did not remain significant after applying Tukey’s post hoc test.

##### Ratio of Time in Open Arms

Using paired *t*-tests, we identified statistically significant differences in the pre- and post-intervention open-arm time ratio within the Khat group (*p* < 0.05) and the Khat-CA-DHK group (*p* < 0.01) (Figure 3B) (Control, t = 1.193 and df = 8; Khat, t = 2.528 and df = 8; Khat-CA, t = 0.4397 and df = 8; Khat-CA-DHK, t = 4.362 and df = 7). Although we did not find significant changes in the open-arm time ratio between groups using a one-way ANOVA in the baseline phase (F (3, 31) = 0.8569), the analysis identified a statistically significant between-group difference in the post-intervention phase (F (3, 31) = 3.323, *p* < 0.05), but the difference did not remain significant after applying Tukey’s post hoc test.

##### Total Time in Closed Arms

**Figure 4:**
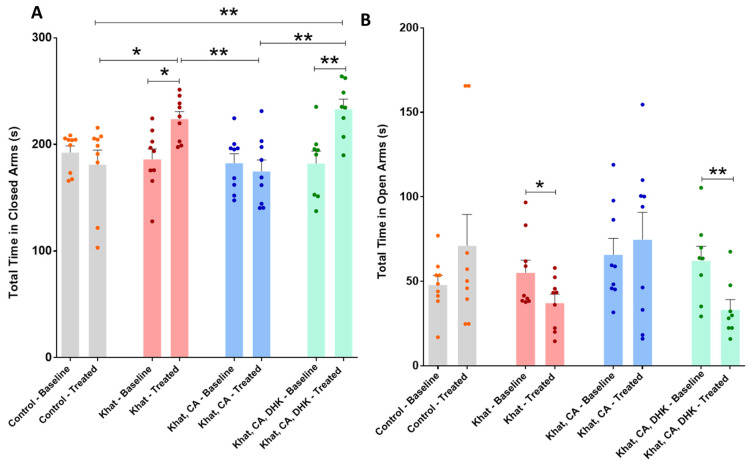
Total time spent in closed and open arms. (**A**) We found significant differences in the total time spent in closed arms between pre- and post-intervention for the Khat (* *p* = 0.0122) and Khat-CA-DHK (** *p* = 0.0016) groups using paired *t*-tests. In the same panel, significant post-intervention differences in the total time spent in closed arms between the Control and Khat (* *p* < 0.05), Khat-CA and Khat groups (** *p* < 0.01), Control and Khat-CA-DHK groups (** *p* < 0.01), and between Khat-CA and Khat-CA-DHK groups (** *p* < 0.01) were identified using one-way ANOVA followed by Tukey’s test. (**B**) We found significant differences in the total time spent in open arms between pre- and post-intervention for the Khat (* *p* = 0.0492) and Khat-CA-DHK (** *p* = 0.0092) groups using paired *t*-tests. In the same panel, we did not identify significant differences in the total time spent in open arms between groups using one-way ANOVA. *n* = 8–9/group. Color dots represent individual data. CA, clavulanic acid; DHK, dihydrokainic acid; s, second.

The total time mice spent in closed arms before and after intervention was compared using paired *t*-tests, which revealed statistically significant within-group differences for the Khat (*p* < 0.05) and Khat-CA-DHK groups only (*p* < 0.01) (Figure 4A) (Control, t = 0.8049 and df = 8; Khat, t = 3.220 and df = 8; Khat-CA, t = 0.5647 and df = 8; Khat-CA-DHK, t = 4.964 and df = 7). We did not find between-group differences in baseline values (F (3, 31) = 0.2841) using a one-way ANOVA but did identify statistically significant differences in post-intervention values (F (3, 31) = 8.045, *p* < 0.001). Post hoc comparisons using Tukey’s test indicated the total time spent in closed arms to be higher in the Khat and Khat-CA-DHK groups as compared with the Control and Khat-CA groups (Figure 4A).

##### Total Time in Open Arms

The total time mice spent in open arms before and after intervention was compared using paired *t*-tests, which showed a statistically significant difference within the Khat group (*p* < 0.05) and the Khat-CA-DHK group (*p* < 0.01) (Figure 4B) (Control, t = 1.338 and df = 8; Khat, t = 2.316 and df = 8; Khat-CA, t = 0.5220 and df = 8; Khat-CA-DHK, t = 3.561 and df = 7). However, we did not identify any statistically significant between-group difference in either pre- (F (3, 31) = 1.027) or post-intervention (F (3, 31) = 2.759, *p* = 0.0588) using a one-way ANOVA.

#### 2.2.3. Novel Object Recognition Test (NORT)

The NORT was used to investigate the effects of khat extract and treatments on memory function.

##### Novel Object Recognition Test (Discrimination Index)

**Figure 5:**
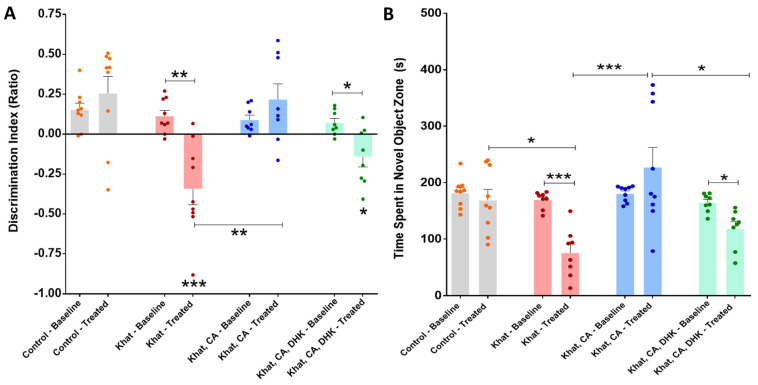
Novel object recognition test. (**A**) We found significant differences in the pre- and post-intervention discrimination index for the Khat (** *p =* 0.0017) and Khat-CA-DHK (* *p =* 0.0157) groups using paired *t*-tests. In the same panel, we identified significant post-intervention differences in the discrimination index between the Control and Khat (*** *p* < 0.001), Control and Khat-CA-DHK (* *p* < 0.05), and between Khat-CA and Khat (** *p* < 0.01) groups using one-way ANOVA followed by Tukey’s test. (**B**) We reported significant differences in the pre- and post-intervention time spent with a novel object for the Khat (*** *p* = 0.0002) and Khat-CA-DHK (* *p* = 0.0145) groups using paired *t*-tests. In the same panel, significant post-intervention differences in the time spent in novel object zone between the Control and Khat (* *p* < 0.05), Khat-CA and Khat groups (*** *p* < 0.001), and Khat-CA and Khat-CA-DHK groups (* *p* < 0.05) were identified using one-way ANOVA followed by Tukey’s test. *n* = 8–9/group. The significance symbols were placed just above any group’s bar when it was compared to the Control group for discrimination index. Color dots represent individual data. CA, clavulanic acid; DHK, dihydrokainic acid; s, second.

Using paired *t*-tests, we found a statistically significant difference in the pre- and post-intervention discrimination index for the Khat group (*p* < 0.01) and the Khat-CA-DHK group (*p* < 0.05) (Figure 5A) (Control, t = 0.9383 and df = 8; Khat, t = 4.616 and df = 8; Khat-CA, t = 1.075 and df = 7; Khat-CA-DHK, t = 3.172 and df = 7). Additionally, we identified the mean discrimination index to be significantly different among all groups after the intervention (F (3, 30) = 9.764, *p* < 0.001) using a one-way ANOVA, but not in the baseline phase (F (3, 30) = 1.073). After applying post hoc comparisons using Tukey’s multiple comparison test, we reported a statistically significant lower discrimination index in the Khat and Khat-CA-DHK groups as compared with the Control and between Khat and Khat-CA groups (Figure 5A).

##### Time Spent in the Novel Object Zone

A statistically significant differences between pre- and post-intervention time spent in the novel object zone within the Khat group (*p* < 0.001) and the Khat-CA-DHK group (*p* < 0.05) were found using paired *t*-tests (Figure 5B) (Control, t = 0.8555 and df = 8; Khat, t = 7.180 and df = 7; Khat-CA, t = 1.307 and df = 8; Khat-CA-DHK, t = 3.227 and df = 7). Similarly, using a one-way ANOVA, we found the mean time spent with a novel object to be significantly different among the four groups after the intervention (F (3, 30) = 7.956, *p* < 0.001), but not in the baseline phase (F (3, 30) = 1.696). Statistically significant differences were found between the Control/Khat, Khat/Khat-CA, and Khat-CA/Khat-CA-DHK groups using post hoc Tukey’s comparisons test (Figure 5B).

### 2.3. Western Blot Analysis

#### 2.3.1. GLT-1 Expression

**Figure 6:**
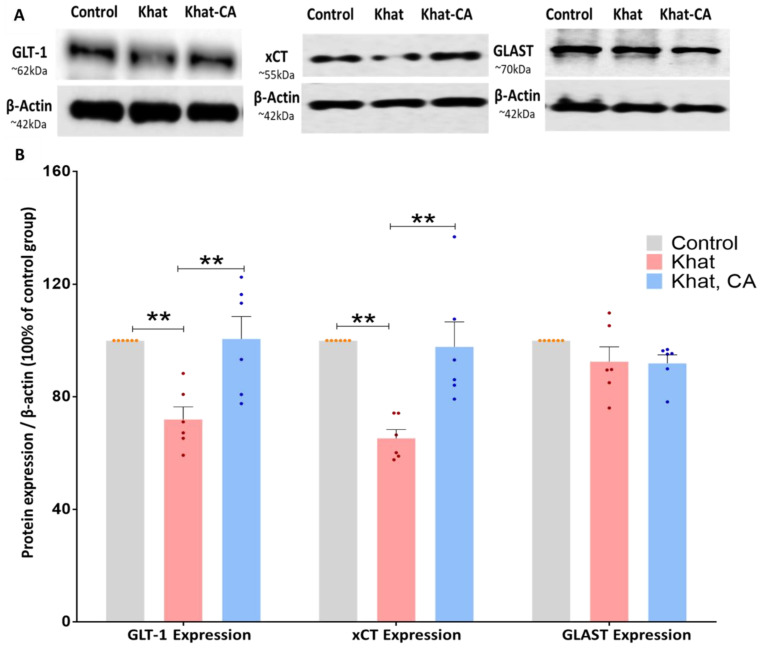
Western blotting of proteins in the nucleus accumbens (NAc); expression relative to β-actin. (**A**) Blot image showing bands of the proteins of interest and β-actin. (**B**) Using one-way ANOVA followed by Tukey’s test, we found GLT-1 and xCT expression to be significantly higher in the Khat group as compared with controls and also in the Khat-CA group as compared with the Khat group. Using one-way ANOVA, we did not identify any significant differences in GLAST expression. Color dots represent individual data. ** *p* < 0.01. *n* = 6/group. CA, clavulanic acid.

We investigated GLT-1 expression in mice treated with khat extract with and without also receiving clavulanic acid treatment. We found a statistically significant difference between the groups (F (2, 15) = 9.871, *p* = 0.0018) using a one-way ANOVA. Subsequent post-hoc analysis using Tukey’s multiple comparison test, we reported significant differences to exist between the Khat group and those that received khat and clavulanic acid (*p* < 0.01), as well as between the Control group and the Khat group (*p* < 0.01) (Figure 6).

#### 2.3.2. xCT Expression

Similarly to GLT-1, xCT expression in the NAc was evaluated by Western blot in mice treated with khat extract with/without clavulanic acid. We found a statistically significant difference between the groups (F (2, 15) = 13.13, *p* = 0.0005) using a one-way ANOVA. After applying post hoc analysis with Tukey’s multiple comparison test, we identified significant differences between the Khat group and those that received khat and clavulanic acid (*p* < 0.01), as well as between the Control group and the Khat group (*p* < 0.01) (Figure 6).

#### 2.3.3. GLAST Expression

We further investigated the effect of khat extract on GLAST protein expression in mice treated with khat alone or khat with clavulanic acid. Using a one-way ANOVA, we identified no significant differences between groups (F (2, 15) = 1.703, *p* = 0.2156) (Figure 6).

### 2.4. Gas Chromatography-Mass Spectrometry (GC-MS)

**Figure 7:**
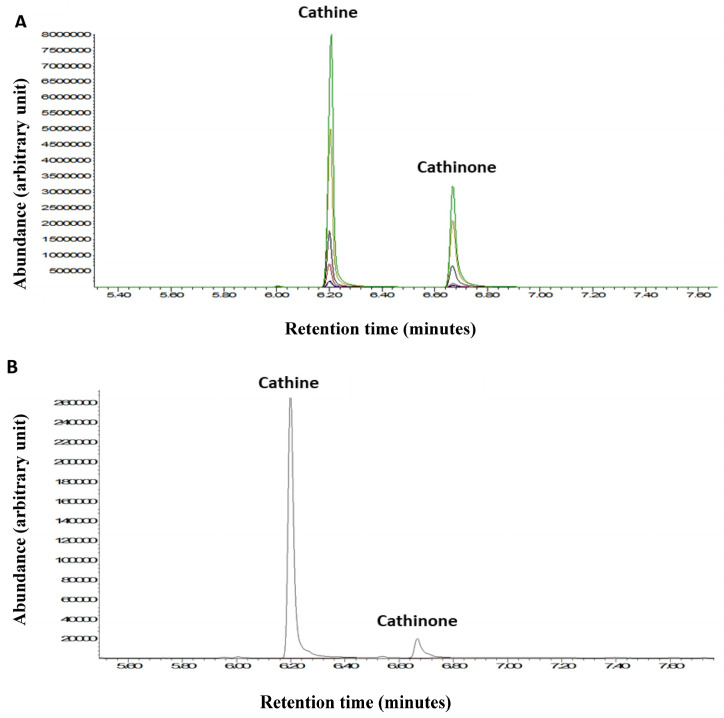
(**A**) GC-MS chromatogram of the cathine and cathinone standards. (**B**) Cathine and cathinone peaks from a khat extract sample. Different colors represent different standard concentrations.

**Figure 8:**
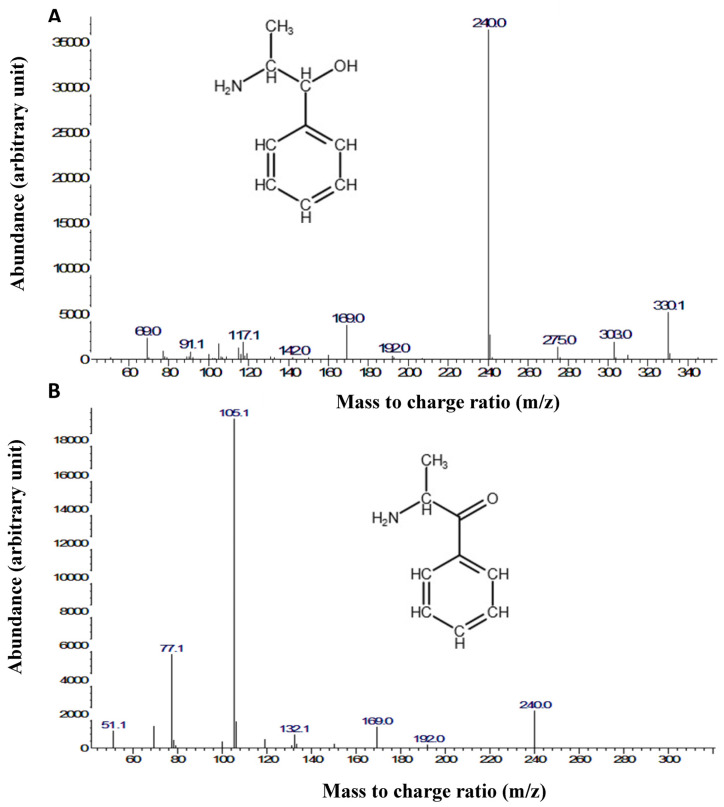
Mass spectra and chemical structures of (**A**) cathine and (**B**) cathinone.

GC-MS analysis was used to quantify the two major psychostimulant active ingredients, cathine and cathinone, in the khat extract used in the present study. This detection was performed with reference to external standards of different known concentrations of cathine and cathinone (Figure 7A). The peaks of the cathine and cathinone in the khat extract are shown at retention times’ of ~6.2 and ~6.7 min, respectively (Figure 7B). The mass spectra of cathine and cathinone are illustrated in Figure 8.

## 3. Discussion

The use of khat extract is reported to have both physical effects, such as weight loss, and behavioral effects, such as increased locomotor activity and anxiety. These effects may be attributed, at least in part, to impaired uptake of the excitatory neurotransmitter glutamate. In the current work, we hypothesized that the β-lactam compound clavulanic acid, which upregulates the glutamate transporter GLT-1, could modulate the neurotoxic and neurobehavioral changes associated with repeated khat extract exposure in mice.

Regarding physical effects, a previous study found body mass index to be lower in khat users as compared to controls, although lean body mass was not affected [42]. In the present study, we observed a non-significant decrease in average mouse body weight over days 1 through 10 of khat exposure; however, weights returned to baseline levels in the last week of the study. This is consistent with a meta-analysis that determined the results of preclinical research studies (up to 2017) investigating the effects of khat on obesity are insufficient to be translated to clinical research [43]. It has also been reported that while khat may reduce hunger, it does not affect the levels of ghrelin and peptide YY, suggesting that the effect of khat on hunger is secondary to the effect of cathinone on CNS pathways [44]. While both animal research and human studies support that khat reduces hunger, there remains limited information on the mechanism and the consequences in terms of weight loss [42,45]. Factors such as age, strain, gender and whether the khat extract is old or fresh may play a role in the average body weight.

Khat extract has been demonstrated to increase locomotor activity. However, in the present study, repeated administration of khat to C57BL/6 mice did not affect locomotor activity (measured as the total distance traveled). Kimani et al. performed daily i.p. injections on mice with khat for 17 days [46] and found the effect to be dose-dependent, with the highest dose resulting in the most significant activity increase. Oyungu et al. compared the effects of immediate and delayed khat extract in Sprague Dawley rats and revealed that delayed khat extract requires higher doses to affect locomotor activity [47]. Thus, the lack of evident locomotor effect in the present findings may be attributed to factors such as strain and animal differences, the dose administered, the concentrations of cathine and cathinone in the extract, the extraction method, and the time of extraction after plant collection and preparation.

Regarding behavioral effects, the EPM test was used to evaluate anxiety-related behavior in mice receiving four different treatments, with the ratio of time spent in closed vs. open arms taken as indicative of anxiety. The results showed repeated administration of khat extract to induce anxiety-like behaviors, an effect that was abolished with seven days of clavulanic acid treatment (5 mg/kg, i.p.). Additionally, the effect of clavulanic acid was prevented by additional treatment with DHK, suggesting GLT-1 to be involved in this attenuation. Bedada et al. showed a similar increase in anxiety behaviors among mice receiving 200 mg/kg of khat extract [48], and another study reported cathinone treatment at doses of 1.25 and 2.5 mg/kg for 14 days to induce anxiety-like behaviors [49]. Mohebbi et al. likewise used a rat model to evaluate the effect of clavulanic acid on alcohol withdrawal symptoms, including anxiety. Ethanol (10% *v*/*v*, 2 g/kg) and clavulanic acid (10, 20, 40, 80 mg/kg) were given simultaneously by oral gavage for ten days, after which EPM tests showed all groups receiving clavulanic acid to enter open arms more often and spend more time in them, supporting a positive effect of clavulanic acid on drug-induced anxiety behaviors [50].

We also used the NORT to assess memory defects in mice receiving repeated administration of khat extract, in which test greater discrimination index and more time spent in the novel object zone are considered to indicate improved memory. Statistically significant changes in discrimination ratio and time spent with the novel object were observed in the Khat group after 17 days of exposure. This memory dysfunction was rescued by a 7-day treatment with clavulanic acid, which effect was, in turn, inhibited by DHK. These findings support that repeated administration of khat impairs memory recognition compared with the baseline state and that this effect may be mediated through GLT-1, which is upregulated by clavulanic acid. In a similar study, Limenie et al. investigated the effects of sub-chronic khat extract exposure on learning and memory in Swiss albino rats [51]. The authors found learning and memory to be impaired in the treated rodents, as evaluated by the Morris water maze task. In humans, impaired working memory function among chronic khat users has been demonstrated using the Digit Symbol Substitution Test [52]. Together with our study, these findings support that clavulanic acid treatments might normalize the memory dysfunction induced by khat extract, and this effect may be mediated by GLT-1.

We next evaluated the effect of repeated exposure to khat extract with/without clavulanic acid treatment on GLT-1 expression in the mouse NAc. Repeated administration of khat extract resulted in downregulation of GLT-1, while clavulanic acid treatment restored its expression. The observed khat-induced downregulation of GLT-1 is consistent with previous research showing that withdrawal from chronic exposure to MDPV, a synthetic cathinone, reduces GLT-1 expression in the NAc [39]. Moreover, downregulation of GLT-1 in the NAc has also been observed in animals exposed to ethanol, methamphetamine, and hydrocodone, while treatment with β-lactam compounds attenuates drugs-seeking behaviors and increases GLT-1 expression [28,30,33]. It was demonstrated that clavulanic acid upregulated GLT-1 and xCT protein expression in the NAc in an alcohol animal model [41]. Thus, our finding that clavulanic acid can normalize behavioral changes induced by khat exposure, at least in part by upregulating GLT-1 expression, is consistent with the literature.

We further investigated the effect of repeated khat exposure and clavulanic acid treatment on xCT expression in the mouse NAc. The results showed repeated khat administration to decrease xCT expression and clavulanic acid treatment to reverse this effect. These findings concur with a number of preclinical studies that demonstrated xCT expression in the NAc to be decreased in animals exposed to drugs of abuse such as methamphetamine, cocaine and hydrocodone, and furthermore showed β-lactam compounds to decrease behavioral symptoms and restore xCT expression [30,33,53]. It is important to note that xCT is responsible for modulating extracellular glutamate levels via non-vesicular release in the rodent brain; in fact, this antiporter is responsible for the glutamate released outside of synapses [54,55]. It also functions in exchanging glutamate for cystine from presynaptic neurons via communication with mGluR2/3, which process is critical in restoring glutamate release capability [19,22].

To further characterize the effects of khat extract on astroglial glutamate transporters, we analyzed protein expression of the GLAST glutamate-aspartate transporter in mice treated with khat extract with/without clavulanic acid treatment. This revealed no significant difference between any of the experimental groups. Prior studies have likewise reported that exposure to hydrocodone, cocaine, or methamphetamine does not alter GLAST expression in the mesocorticolimbic system [30,33,53]. These results suggest that expression of GLT-1 and xCT in the NAc is more sensitive to drugs of abuse compared with GLAST expression. This may be due to GLT-1 and xCT being responsible for regulating the majority of glutamate in the brain (~90%).

## 4. Materials and Methods

### 4.1. Materials

Khat was provided by Jazan University, Jazan, Saudi Arabia. It was prepared and extracted using a specific apparatus according to a procedure previously described by Richardson and Harborne [56]. Briefly, 1 kg of khat leaves was minced and macerated in 2.5 L of 80% aqueous ethanol (*v*/*v*) for 72 h at room temperature with continuous shaking. The supernatant was filtered through filter paper (0.45 µm) twice, and the obtained extract was allowed to dry at room temperature. It was then weighed, and the percent yield was determined (10%). The extract was finally refrigerated at 4 °C in dark bottles until further use [56].

Clavulanic acid (MedChemExpress, Monmouth Junction, NJ, USA), dihydrokainic acid (DHK) (SIGMA Life Science, Rolla, MO, USA), and physiological normal saline (0.9%) were prepared daily in the lab. For Western blots, 8–15% SDS-PAGE gels, 3% non-fat milk in Tris-buffered saline with 0.1% Tween^®^ 20 detergent (TBST), and rabbit primary antibodies (anti-GLT-1, anti-xCT, anti-GLAST) were purchased from ABclonal, Wuhan, China. The housekeeping antibody, rabbit anti-β-actin, was also purchased from ABclonal, Wuhan, China.

### 4.2. Experimental Models

Forty male C57BL/6 mice aged 5–8 weeks and weighing 20–25 g were housed in standard group cages and kept under a 12 h light/dark cycle. The mice were obtained from the Experimental Animal Care Centre, College of Pharmacy, King Saud University, Riyadh, Saudi Arabia. The mice were kept in a room with a humidity of 40 to 60% and a temperature of 22 ± 2 °C. Water ad libitum and standard rodent chow were provided to the mice. Injections were given intraperitoneally (i.p.) according to the treatment group assigned. The IACUC committee at King Saud University approved all protocols and procedures for the animal study (KSU-SE-22-53).

### 4.3. Instrumentation

An elevated plus-shaped maze with a 45 × 60 cm Plexiglass box was used to assess animal performance. The maze was monitored by a camera connected to a computer, with the ANY-maze software (version 6) used for recordings. Open field boxes (20 × 40 cm, Plexiglass) connected to activity monitoring software (ANY-maze, version 6) were also used to assess the distance traveled by each mouse. Other equipment included transfer chambers, an imaging system (ChemiDocTMMP Bio-Rad, Hercules, CA, USA), a cryostat machine for brain dissection, and a spectrometer for protein assay.

### 4.4. Study Design

**Figure 9:**
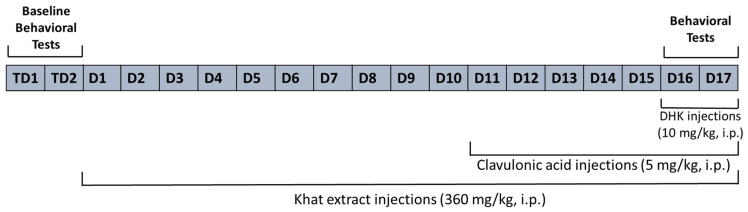
Study timeline. TD, Test day; D, Day.

The study design and timeline are summarized in Figure 9. The mice were divided into four groups, with ten mice per group.

1. The Control group was treated with the vehicle solution (0.9% normal saline) for all 17 days.
2. The Khat group was given khat extract (360 mg/kg, i.p.) for all 17 days [46].
3. The Khat-CA group was given khat extract (360 mg/kg, i.p.) for 17 days and treated with clavulanic acid (5 mg/kg) [33,41] for the last seven days of the study.
4. The Khat-CA-DHK group was given khat extract (360 mg/kg, i.p.) for 17 days and treated with clavulanic acid (5 mg/kg) for the last seven days of the study; in addition, they were given DHK (10 mg/kg, i.p.) [57,58] 30 min before the behavioral experiments.

### 4.5. Behavioral Experiments

Behavioral tests were performed before the start of treatment and on the 16th and 17th days of the experimental procedure for all experimental groups.

#### 4.5.1. Locomotion Test

The open field activity monitoring system comprehensively assesses mouse locomotor and behavioral activity levels and is used for the assessment of locomotive impairment in animal models of neuromuscular disease. As described previously [59], the mice were habituated for 10 min, then placed in open field boxes and allowed to explore for 10 min. During this period, many measures can be tabulated and reported, including the distance moved, time spent moving, instances of rearing, and changes in activity over time. For this study, the distance moved was detected using activity monitoring software.

#### 4.5.2. Elevated Plus Maze (EPM)

The elevated plus maze (EPM) is used to assess anxiety-related behavior in rodent models. The EPM apparatus is a “+”-shaped maze elevated above the floor with two oppositely positioned closed arms, two oppositely positioned open arms, and a central area. The rodent is monitored, and the number of entries and the time spent in each segment are measured. It is perceived that the more relaxed a rodent is, the more willing it is to explore “open” spaces and become exposed; conversely, the more stressed the animal, the more it hides in “closed” spaces [60].

The test was blindly performed during the animal’s light cycle, and each mouse was first habituated for 15 min then monitored for 5 min. We calculated the ratio of time in closed arms by dividing the time spent in closed arms by the total time spent in closed and open arms (Equation (1)). Similarly, the ratio of time in open arms was calculated by dividing the time spent in open arms by the total time spent in closed and open arms (Equation (2)). We also considered the raw time spent in open arms and in closed arms. Animals’ tracking and monitoring were performed using ANY-maze software.

(1) $$Ratio\;in\;closed\;arm=\frac{time\;spent\;in\;closed\;arms}{total\;time\;spent\;in\;closed\;and\;open\;arms}$$

(2) $$Ratio\;in\;open\;arm=\frac{time\;spent\;in\;open\;arms}{total\;time\;spent\;in\;closed\;and\;open\;arms}$$

#### 4.5.3. Novel Object Recognition

The novel object recognition test (NORT) was used as described previously to characterize mouse learning and memory [61,62]. The test was blindly performed during the animal’s light cycle, with each mouse first being habituated for 30 min. For the test, the mouse was placed in an empty box measuring 45 × 60 cm for 5 min and then returned to its home cage. Subsequently, two identical objects were placed in the box, 5 cm from the edge and 5 cm apart, and the mouse was allowed to explore them for 5 min. Then, on the second day, one of the familiar objects was replaced with a novel object, and the mouse was returned to the box for 5 min and allowed to explore both objects. The discrimination index for novel object recognition was calculated by first determining the difference in time spent exploring the novel versus the familiar object, then dividing it by the total time spent with the objects (Equation (3)). The novel object zone is the area around the novel object that is identified by ANY-maze software.

(3) $$Discrimination\;index=\frac{\left[time\;spent\;with\;novel\;object-time\;spent\;with\;familiar\;object\right]}{\left[time\;spent\;with\;novel\;object+time\;spent\;with\;familiar\;object\right]}$$

### 4.6. Brain Harvesting

Mice were euthanized approximately 24 h after the last treatment. The brains were immediately extracted and snap-frozen in a −80 °C freezer for use in immunoblotting. Subsequently, the NAc (~1 mm thickness) was dissected (freehand) using a cryostat machine maintained at −20 °C. Six random samples were selected from each group for western blotting assay. Determination of brain region boundaries was made using the Paxinos and Watson atlas [63].

### 4.7. Western Blotting

Western blotting was used to quantify GLT-1, xCT, and GLAST expression in the NAc, as described in our previous work [64]. Briefly, NAc tissue (*n* = 6 mice per group) was homogenized in a lysis buffer containing phosphatase and protease inhibitors. Total protein was quantified using a BCA assay, and then equal amounts were loaded on 8–15% SDS-PAGE gels. After protein separation by electrophoresis, the bands were transferred to membranes using a transfer chamber. Blocking of membranes was performed using 3% non-fat milk in TBST for one hour, after which the membranes were incubated with primary antibodies overnight at 4 °C; the antibodies used were rabbit anti-GLT-1 (1:1000, ABclonal, Wuhan, China), rabbit anti-xCT (1:1000, ABclonal, Wuhan, China), rabbit anti-GLAST (1:1000, ABclonal, Wuhan, China), and rabbit anti-actin (1:1000, ABclonal, Wuhan, China). On the second day, the membranes were incubated with an anti-rabbit secondary antibody for 90 min. Western blot detection reagents were then added, and the signal was detected with an imaging system (ChemiDocTMMP Bio-Rad, Hercules, CA, USA). Image J software (Java 8) was used to quantitatively measure the expression of the proteins of interest. All treatment groups were normalized to the expression of the Control group in the same gel. In order to identify significant changes in the expression of the proteins (compared to the water control group) in the NAc, the data for the water control group were expressed as 100% as performed in previous studies [30,33,53,65,66,67,68]. As a result, we compared the expression of all the relevant proteins in each treatment group to that of the water control group, which was also run on the same gel set.

### 4.8. Gas Chromatography-Mass Spectrometry

Gas chromatography-mass spectrometry (GC-MS) was used to identify cathine and cathinone, two of the major psychostimulant active ingredients in khat extract, as described in a previous study [69]. External standards of cathine and cathinone were obtained from Naif Arab University for Security Sciences and used to establish the methodology of cathine and cathinone identification via GC-MS. Briefly, the khat extract was first dissolved in ethyl acetate, and heptafluorobutyric anhydride was added to the mixture for sample derivatization. Samples were then sealed and incubated in a heat block for 30 min at 65–70 °C, allowed to cool to room temperature, then dried by evaporation under a stream of nitrogen. Finally, they were reconstituted in ethyl acetate and injected into the GC-MS system.

### 4.9. Statistical Analysis

All data were analyzed using PRISM software version 6.01. Behavioral data from the locomotion, novel object recognition, and EMP tests were evaluated using paired *t*-tests to compare the obtained data before and after treatments for each parameter in each group. Differences between the groups in the baseline and post-treatment periods were evaluated by a one-way ANOVA followed by Tukey’s post hoc comparison test. Protein expression of GLT-1, xCT, and GLAST was first normalized by corresponding β-actin expression and then to the Control group, after which differences were evaluated using a one-way ANOVA followed by Tukey’s post hoc test. Differences in mouse weight were assessed through a two-way ANOVA. Results were deemed statistically significant when *p* ≤ 0.05. GraphPad Prism was used to calculate the outliers, and these outliers were excluded from the analysis.

## 5. Conclusions

**Figure 10:**
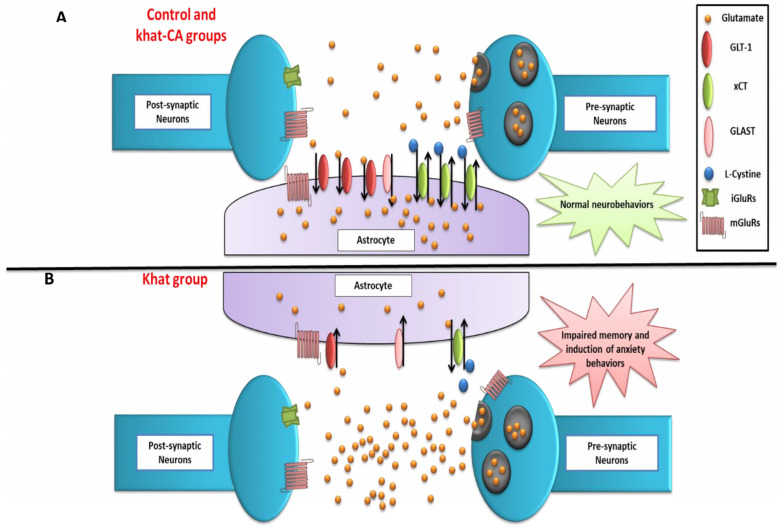
Schematic diagram summarizing the effects of khat treatment and rescue by clavulanic acid on neurobehaviors, nucleus accumbens GLT-1 and xCT expression, and extracellular glutamate concentrations. (**A**) Control and Khat-CA groups. (**B**) Khat group. The effects of treatments on other markers investigated in other studies are not shown. CA, clavulanic acid; GLT-1, glutamate transporter 1; xCT, glutamate/cystine antiporter; GLAST, glutamate-aspartate transporter; iGluRs, ionotropic glutamate receptors; mGluRs, metabotropic glutamate receptors.

Because the majority of GLT-1 proteins are expressed in astrocytes, we hypothesized here that GLT-1 and xCT expression in the astrocytes in the NAc are decreased and consequently led to altered glutamatergic system and impaired neurobehavioral effects in mice repeatedly exposed to khat (Figure 10). Future studies are warranted to perform immunohistochemistry techniques to study the glutamatergic transporter in the NAc using a similar study design to confirm our hypothesis. The use of β-lactam compounds, such as clavulanic acid, significantly reverses the downregulation of glutamate transporters and positively normalizes the neurobehavioral changes associated with repeated exposure to khat in this preclinical model (Figure 10). Future studies are warranted to conduct a detailed genetic analysis at the neurological interface, which would complement the protein and behavioral studies. In addition, drug development studies can take clavulanic acid as a starting point to discover new β-lactam compounds with optimal safety and efficacy profiles for potential use as therapeutic compounds in treating substance use disorders.

## Data Availability

Available upon request.
